# Multi-Year Variation of Ozone and Particulate Matter in Northeast China Based on the Tracking Air Pollution in China (TAP) Data

**DOI:** 10.3390/ijerph19073830

**Published:** 2022-03-23

**Authors:** Hujia Zhao, Ke Gui, Yanjun Ma, Yangfeng Wang, Yaqiang Wang, Hong Wang, Yu Zheng, Lei Li, Lei Zhang, Yuqi Zhang, Huizheng Che, Xiaoye Zhang

**Affiliations:** 1 Institute of Atmospheric Environment, China Meteorological Administration, Shenyang 110166, China; mayanjun@iaesy.cn (Y.M.); wangyangfeng@iaesy.cn (Y.W.); 2 State Key Laboratory of Severe Weather (LASW), Key Laboratory for Atmospheric Chemistry (LAC), Institute of Atmospheric Composition and Environmental Meteorology, Chinese Academy of Meteorological Sciences (CAMS), CMA, Beijing 100081, China; guik@cma.gov.cn (K.G.); yqwang@cma.gov.cn (Y.W.); wangh@cma.gov.cn (H.W.); yuzheng@cma.gov.cn (Y.Z.); lilei@radi.ac.cn (L.L.); leiz09@cma.gov.cn (L.Z.); chehz@cma.gov.cn (H.C.); xiaoye@cma.gov.cn (X.Z.); 3 Liaoning Meteorological Service Center, Liaoning Meteorological Bureau, Shenyang 110166, China; caihongke1984@163.com

**Keywords:** O_3_-8h-90per, PM_2.5_, distribution, interannual, city level, Northeast China

## Abstract

With the rapid development of economy and urbanization acceleration, ozone (O_3_) pollution has become the main factor of urban air pollution in China after particulate matter. In this study, 90th percentile of maximum daily average (MDA) 8 h O_3_ (O_3_-8h-90per) and PM_2.5_ data from the Tracking Air Pollution in China (TAP) dataset were used to determine the mean annual, seasonal, monthly, and interannual distribution of O_3_-8h-90per and PM_2.5_ concentrations in Northeast China (NEC). The O_3_-8h-90per concentration was highest in Liaoning (>100 μg/m^3^), whereas the highest PM_2.5_ concentration was observed mainly in urban areas of central Liaoning and the Harbin–Changchun urban agglomeration (approximately 60 μg/m^3^). The O_3_-8h-90per concentrations were highest in spring and summer due to more intense solar radiation. On the contrary, the PM_2.5_ concentration increased considerably in winter influenced by anthropogenic activities. In May and June, the highest monthly mean O_3_-8h-90per concentrations were observed in central and western Liaoning, about 170–180 μg/m^3^, while the PM_2.5_ concentrations were the highest in January, February, and December, approximately 100 μg/m^3^. The annual mean O_3_-8h-90per concentration in NEC showed an increasing trend, while the PM_2.5_ concentration exhibited an annual decline. By 2020, the annual mean O_3_-8h-90per concentration in southern Liaoning had increased considerably, reaching 120–130 μg/m^3^. From the perspective of city levels, PM_2.5_ and O_3_-8h-90per also showed an opposite variation trend in the 35 cities of NEC. The reduced tropospheric NO_2_ column is consistent with the decreasing trend of the interannual PM_2.5_, while the increased surface temperature could be the main meteorological factor affecting the O_3_-8h-90per concentration in NEC. The results of this study enable a comprehensive understanding of the regional and climatological O_3_-8h-90per and PM_2.5_ distribution at distinct spatial and temporal scales in NEC.

## 1. Introduction

Surface ozone (O_3_) is a critical gaseous pollutant that affects air quality and contributes to climate change [1,2,3,4]. O_3_ and its interactions with precursor gaseous emissions have been widely researched in recent decades [5,6]. Surface O_3_ is formed by photochemical reactions of volatile organic compounds (VOCs) and nitrogen oxides; these reactions are exacerbated by sunlight [7,8,9]. The major contributions to O_3_ formation and accumulation emanate from natural emissions and anthropogenic sources such as long-range transport [10,11,12,13]. Worldwide, numerous studies have analyzed the relationship between surface O_3_ and temperature, pressure, relative humidity, wind, precipitation, cloud cover, and solar radiation, as well as the meteorological factors related to synoptic circulation patterns [14,15,16,17]. 

Due to several decades of rapid economic growth and urbanization, China has experienced severe fine particulate matter (PM_2.5_) pollution originating from anthropogenic emissions [18,19,20]. However, in recent years, O_3_ has become a more severe air pollutant than PM_2.5_ is in some Chinese cities, resulting from a substantial increase of O_3_ precursor emissions such as VOCs [21,22,23,24]. Numerous studies have reported the coordinated pollution of O_3_ and particulate matter as the primary urban pollutant in densely populated areas, such as Beijing–Tianjin–Hebei (BTH), the Yangtze River Delta (YRD), the Pearl River Delta (PRD), and the Sichuan Basin [25,26,27]. Zeng et al. (2020) [28] implied that the concentrations of PM_2.5_ and O_3_ showed different seasonal trends in eastern China. Liu et al. (2021) [29] used the ground-based hyperspectral stereoscopic remote sensing network to provide a promising strategy to support management of PM_2.5_ and O_3_ and their precursors and conduct attribution of sources. In the formation of surface O_3_, meteorological factors play a crucial role [30,31,32,33,34]. Yang et al. (2021) [35] indicated that PM_2.5_ was primarily affected by wind, temperature, and rainfall, while O_3_ was mostly influenced by temperature, relative humidity, and sunshine duration in 284 major cities in China. Dai et al. (2020) [36] identified that the number of days with co-pollution of O_3_ and PM_2.5_ was mainly dependent on relative humidity, surface air temperature, and wind speed in the Yangtze River Delta region.

**Figure 1:**
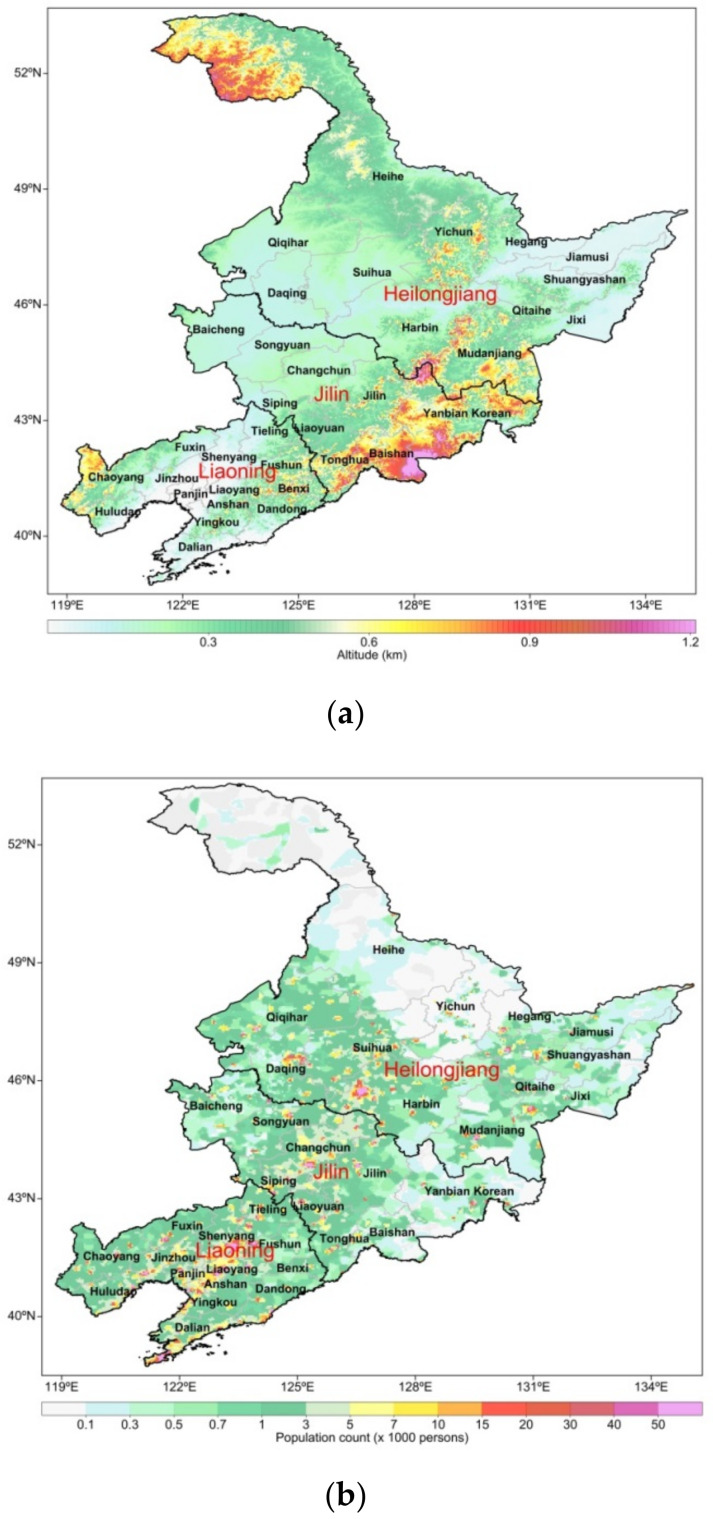
(**a**) Geography and city distribution and (**b**) population density in NEC.

Northeast China (NEC) is a key region in Asia, bordering Siberia in the north, the Bohai and Yellow Seas and the Central Plains in the south, Mongolia to the west, and the Korean Peninsula and the Sea of Japan to the east (Figure 1a). Air quality in NEC has been improving because efforts to control PM_2.5_ have been intensified. However, O_3_ has become a new environmental problem. Due to the unique geographical location and meteorological conditions of NEC, O_3_ pollution originates not only from local emissions but also from those of surrounding areas. Figure 1b shows the spatial distribution of population density in NEC. The NEC has an uneven distribution of population, while southwest and central NEC are densely populated with a maximum population density of nearly 50 (×1000 persons), followed by eastern NEC with a population density of less than 15 (×1000 persons). The northern mountain of NEC is sparsely populated, with a population density of less than 0.5 (×1000 persons). The population density in NEC is mainly affected by topography, climate, and economic development, and the natural factors and social factors simultaneously affect the change of population density. From the spatial distribution of population density in the three provinces of NEC, population density is greater in Liaoning than in Jilin province, and lower in Heilongjiang province. The provincial capital cities mainly present a spatial pattern of polycentric concentrated distribution of population. These results indicate that the level of social and economic development is the main factor influencing the distribution of population density in NEC. Therefore, studies in NEC have revealed the importance of considering both local emission sources as well as the long-range transport of pollutants from regions such as the North China Plain [37,38]. Zhu and Liao (2016) [39] reported that surface O_3_ levels in NEC were comparable to those of BTH and the YRD and even higher than those in the PRD during spring 2000. NEC is a crucial industrial and agricultural base in China. More research on O_3_ pollution and its impact on ecosystem security and agriculture is needed. Understanding the spatiotemporal variation of O_3_ and PM_2.5_ can aid in providing technical support for the prevention and control of O_3_ and PM_2.5_ pollution in NEC. 

This study analyzed the mean annual, seasonal, monthly, and interannual variation and distribution of O_3_-8h-90per and PM_2.5_ in NEC. The innovation in this study was its focus on the multi-year variation and distribution of O_3_-8h-90per and PM_2.5_ in NEC. The objective was to investigate the historical variation in surface O_3_-8h-90per (from 2013 to 2020) and PM_2.5_ (from 2001 to 2020) in NEC on the basis of the Tracking Air Pollution in China (TAP) dataset. The remainder of this paper is structured as follows: Section 2 describes the study area and data sources. Section 3 presents the multi-year variation in O_3_-8h-90per and PM_2.5_ at temporal (annual, seasonal, monthly, and interannual) and spatial (regional and city level) scales. Section 4 and Section 5 discusses and presents the conclusions of this study.

## 2. Materials and Methods

NEC (120°–135° E, 40°–53° N) mainly encompasses Heilongjiang, Jilin, and Liaoning provinces. Liaoning is the most populous province and lies in the south of NEC. To the north lie Jilin and the northernmost province of Heilongjiang, which are the major agricultural provinces in the region. Precipitation is highest in summer; winter is characterized by snowfall, and surface snow usually remains for prolonged periods. NEC is the snowiest region in China. In recent years, the intensification of anthropogenic pollutants and adverse meteorological conditions have resulted in numerous processes causing air pollution. In cities with severe pollution and low air quality, particulate matter and O_3_ have become the primary pollutants [40,41,42].

The TAP dataset was developed at Tsinghua University as a cooperative effort among several institutions and teams [43]. The aim was to build a multiscale, near-real-time aerosol and gaseous pollutant concentration database in China and provide essential support for pollution characteristics analysis. The TAP database was generated using state-of-the-art technology involving machine learning algorithms [44,45]. The TAP data are determined based on the combination of multisource data including ground measurements, satellite aerosol optical parameter retrievals, model simulations, and meteorology field, land use information as well as population, and elevation data by multilayer machine learning models. It integrates real-time ground observations, near-real-time satellite remote sensing information, and air quality model simulation with multisource big data and provides near-real-time data while ensuring complete spatiotemporal coverage. Surface O_3_-8h-90per concentration data (2013–2020) and PM_2.5_ data (2001–2020) for China with a 10 km resolution can be downloaded from http://tapdata.org (last access: 1 July 2021). To our knowledge, this is the first study to use the pollutant concentration data from the newly released TAP dataset for investigating pollution in NEC. In this paper, we define O_3_-8h-90per as the average daily maximum O_3_-8h-90 per at the 90th percentile level. The annual, monthly, and seasonal average of O_3_-8h-90per data was used to analyze the temporal scale and spatial distribution of O_3_ (μg/m^3^), with the minimum value of 1.00 μg/m^3^.

Population data were obtained from the combined datasets of the GPWv3 (Gridded Population of the World available at 5-year intervals from 1990 to 2000) and GPWv4 (available at 5-year intervals from 2000 to 2020), from the NASA Socioeconomic Data and Applications Center (http://sedac.ciesin.columbia.edu/, last accessed on 1 July 2021), and the resolution was 2.5 arcminute.

The monthly mean tropospheric NO_2_ column data from 2005 to 2020 were retrieved from the Ozone Monitoring Instrument (OMI) observations with a spatial resolution of 0.125° and are available from the Tropospheric Emission Monitoring Internet Service (TEMIS; https://www.temis.nl/index.php/, last accessed 1 July 2021). Four key meteorological factors, precipitation (PPT), wind speed (WS), boundary layer height (BLH), and temperature at 2 m (T2) from 2001 to 2020, were obtained from the European Centre for Medium-Range Weather Forecasts (ECMWF) Reanalysis v5 (ERA5; https://cds.climate.copernicus.eu/cdsapp#!/search?type=dataset/, last access: 1 July 2021) with a spatial resolution of 0.25° × 0.25°.

## 3. Results

### 3.1. Annual Distribution of O_3_-8h-90per and PM_2.5_ in NEC

**Figure 2:**
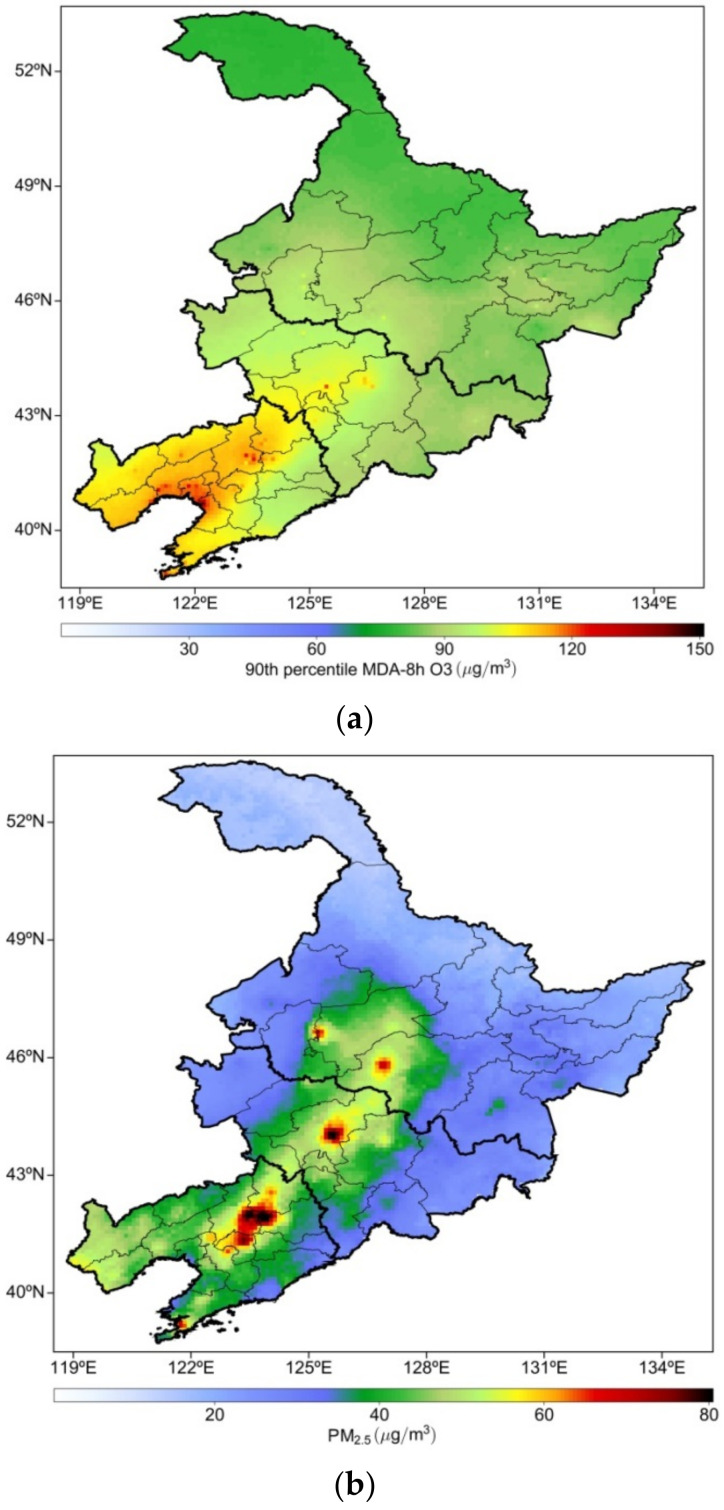
Spatial distribution of mean annual concentrations of (**a**) O_3_-8h-90per averaged from 2013–2020 and (**b**) PM_2.5_ averaged from 2001–2020 in NEC.

The spatial distribution of the mean annual O_3_-8h-90per concentrations in NEC from 2013 to 2020 is presented in Figure 2a. The O_3_-8h-90per concentration was highest in Liaoning (>100 μg/m^3^); that in northern, western, and southern Liaoning and the Bohai Rim area reached 120 μg/m^3^. By contrast, the O_3_-8h-90per concentration in eastern Liaoning was lower, at approximately 90 μg/m^3^. The O_3_-8h-90per concentration in Jilin was lower than that in Liaoning and distributed in central and western Jilin. The highest O_3_-8h-90per concentration was approximately 90–100 μg/m^3^, and that in other areas of Jilin was approximately 80 μg/m^3^. Compared with the spatial distribution of O_3_-8h-90per concentrations in Liaoning and Jilin, the O_3_-8h-90per concentration in Heilongjiang was relatively low at approximately 80 μg/m^3^. 

Figure 2b depicts the distribution of the multiyear mean concentrations of PM_2.5_ in NEC. Compared with the high O_3_-8h-90per concentration in western Liaoning, the high PM_2.5_ concentration in NEC was distributed mainly in the urban areas of central Liaoning, the Harbin–Changchun urban agglomeration, and other areas where industrial production is expanding and populations growing. Zhao et al. (2021) [46] reported that PM_2.5_ was concentrated mostly in central Liaoning, western Jilin, and Heilongjiang. The highest PM_2.5_ concentration was approximately 60 μg/m^3^ and occurred in Shenyang, Changchun, Harbin, and other provincial capitals of NEC. With approximately 50 μg/m^3^, the PM_2.5_ concentration in western Liaoning was higher than that in other areas. The distribution range of high PM_2.5_ concentrations in western Liaoning was not as large as that of O_3_-8h-90per concentrations. Except for the central cities, the PM_2.5_ concentration in Jilin was approximately 30–40 μg/m^3^, and the lowest PM_2.5_ concentration was approximately 20 μg/m^3^ in the eastern and western marginal areas of Jilin. At approximately 10–20 μg/m^3^, the PM_2.5_ concentration in northern Heilongjiang was relatively low.

The spatial distribution of O_3_-8h-90per (from 2013 to 2020) and PM_2.5_ (from 2001 to 2020) concentrations in NEC was closely related to the environmental background, population density, and meteorological condition. The O_3_-8h-90per pollution in NEC was concentrated mainly in central and western Liaoning, followed by central Jilin; the overall O_3_-8h-90per concentration in Heilongjiang was low. However, high levels of PM_2.5_ were observed throughout the densely populated areas of the three provinces. Due to rapid and geographically distinct urbanization patterns, numerous anthropogenic pollution sources are likely to have led to a regional imbalance in the distribution of PM_2.5_ concentrations. However, the spatial distribution of high PM_2.5_ concentrations was inconsistent with that of high O_3_-8h-90per concentrations. This indicates that although pollutants can increase O_3_ precursors to a certain extent, they are not the main factors affecting the distribution of O_3_-8h-90per concentrations in NEC. In particular, important air pollutants such as volatile organic compounds (VOCs) are also important precursors of PM_2.5_, and various factors affect the correlation between O_3_-8h-90per and PM_2.5_. The existence of a high concentration of O_3_ in the atmosphere enhances oxidation of the atmosphere, which is conducive to the formation of secondary particulate matter and thus an increase in PM_2.5_ pollution. The presence of a large amount of PM_2.5_ weakens the solar radiation reaching the near ground, reducing the photodecomposition reaction rate of O_3_.

### 3.2. Seasonal Distribution of O_3_-8h-90per and PM_2.5_ in NEC

**Figure 3:**
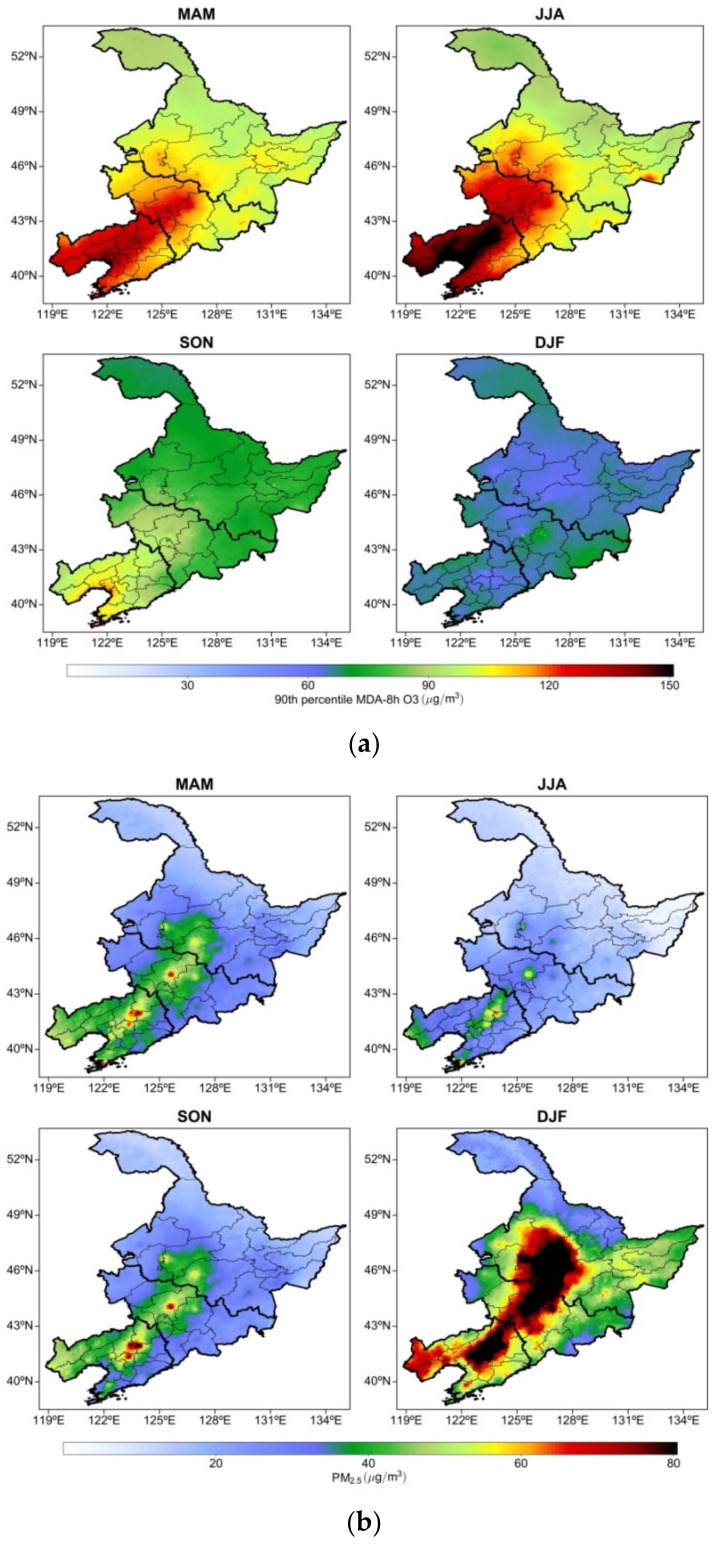
Spatial distribution of mean seasonal concentrations of (**a**) O_3_-8h-90per averaged from 2013–2020 and (**b**) PM_2.5_ averaged from 2001–2020 in NEC.

In this study, the four seasons were spring (March, April, and May), summer (June, July, and August), autumn (September, October, and November) and winter (December, January, and February). The O_3_-8h-90per concentrations were higher in spring and summer, followed by autumn and winter (Figure 3a). In spring, the highest O_3_-8h-90per concentrations, approximately 120–130 μg/m^3^, were observed in central and western Liaoning. At approximately 120 μg/m^3^, the O_3_-8h-90per concentration in central Jilin was relatively high. At approximately 90–100 μg/m^3^, the O_3_-8h-90per concentration throughout Heilongjiang was relatively low. In summer, the O_3_-8h-90per concentrations increased considerably throughout Liaoning, and the highest O_3_-8h-90per concentration was observed in central Liaoning and the Bohai Rim region (150 μg/m^3^). The next-highest values were observed in central and western Jilin (approximately 120 μg/m^3^) and Heilongjiang (approximately 90–100 μg/m^3^). In autumn, the O_3_-8h-90per concentration in NEC decreased markedly. The O_3_-8h-90per concentration in central Liaoning was approximately 100–110 μg/m^3^, and that in Jilin and Heilongjiang decreased to 70–80 μg/m^3^. In winter, the O_3_-8h-90per concentration in NEC reached one of the lowest values of the whole year, approximately 60 μg/m^3^. The O_3_-8h-90per concentrations in some areas of Jilin were relatively high, approximately 70–80 μg/m^3^. Favorable meteorological conditions such as high temperatures and strong solar radiation can contribute to high O_3_-8h-90per concentrations. In addition, the abundant carbon aerosols produced by biomass burning in spring affect the generation of O_3_-8h-90per [47].

Unlike the distribution of O_3_-8h-90per concentrations, that of PM_2.5_ concentrations increased considerably in winter, followed by that in spring and autumn. The lowest PM_2.5_ concentrations were observed in summer (Figure 3b). In spring, the highest PM_2.5_ concentrations were observed in central Liaoning and central Jilin (approximately 60 μg/m^3^). The PM_2.5_ concentration in a typical city reaches 80 μg/m^3^. By contrast, the PM_2.5_ concentration in Heilongjiang was relatively low, approximately 20–30 μg/m^3^. In summer, the PM_2.5_ concentration in NEC decreased. Except for the PM_2.5_ concentrations in some central cities of Liaoning, which exhibited values of approximately 50–60 μg/m^3^, the PM_2.5_ concentration in NEC was lower than 30 μg/m^3^. In autumn, the PM_2.5_ concentration in central Liaoning increased considerably. The highest concentration was approximately 80 μg/m^3^, and the PM_2.5_ concentration in western Liaoning exhibited an increasing trend, reaching approximately 50 μg/m^3^. Similarly, the PM_2.5_ concentration was approximately 50 μg/m^3^ in central Jilin. In autumn, the PM_2.5_ concentrations in most parts of Heilongjiang were low, approximately 20–30 μg/m^3^. In winter, a peak in PM_2.5_ concentration was observed in NEC. The highest PM_2.5_ concentration was approximately 90 μg/m^3^ in central Liaoning, central Jilin, and central as well as western Heilongjiang. The PM_2.5_ concentration in NEC was approximately 50–60 μg/m^3^. By contrast, the PM_2.5_ concentration in northern Heilongjiang was low, approximately 20 μg/m^3^. 

The seasonal distribution of O_3_-8h-90per and PM_2.5_ concentrations in NEC indicates that the PM_2.5_ concentration was the highest in winter (affected mainly by emissions from coal burning), whereas the winter O_3_-8h-90per concentration reached one of the lowest values of the whole year. This indicates that favorable meteorological conditions such as high temperatures and strong solar radiation were crucial factors affecting the formation of O_3_. Zhao et al. (2019) [48] reported that low concentrations of O_3_ were observed during winter (when heating is used), whereas relatively higher concentrations were observed in spring, when longer and stronger solar radiation drives the photochemical processes that lead to the formation of O_3_ and heating is not used. Xia et al. (2021) [49] also found that high values of daytime maximum, nighttime minimum, and diurnal difference of summer ozone concentration occurred in several city agglomerations of northern and southern China.

### 3.3. Monthly Distribution of O_3_-8h-90per and PM_2.5_ in NEC

**Figure 4:**
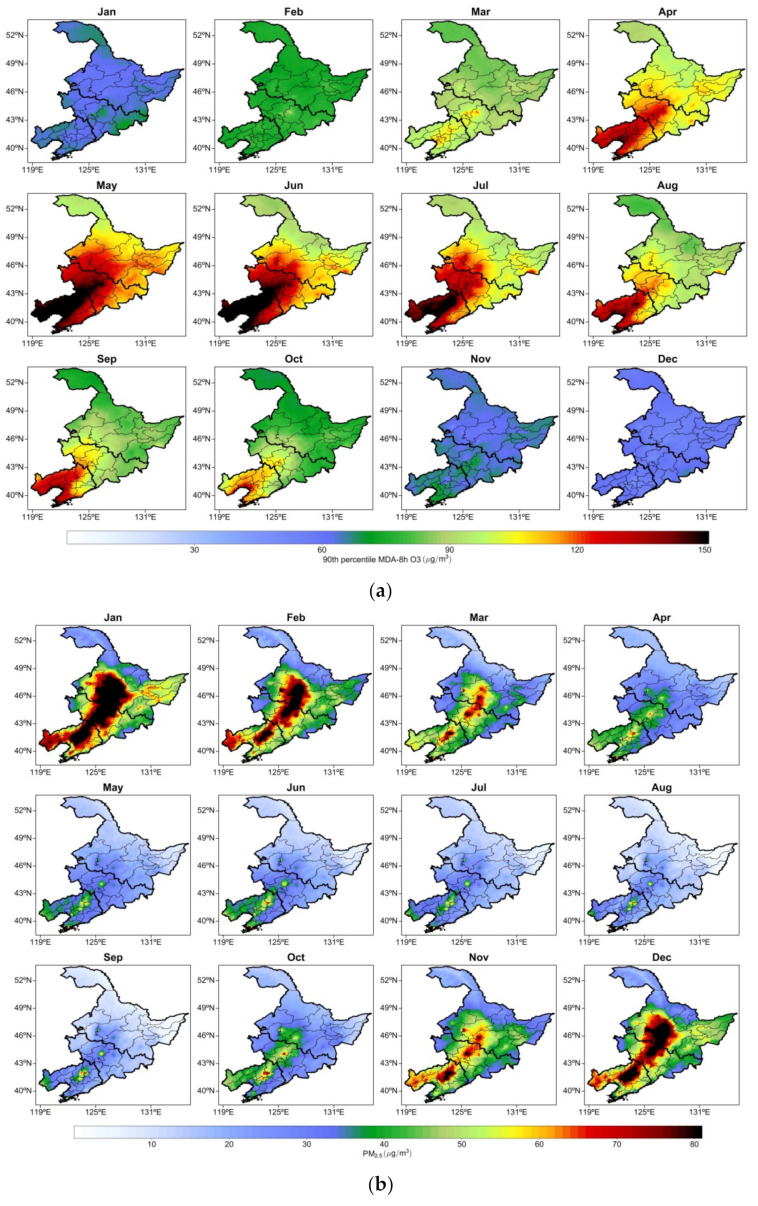
Spatial distribution of mean monthly concentrations of (**a**) O_3_-8h-90per averaged from 2013–2020 and (**b**) PM_2.5_ averaged from 2001–2020 in NEC.

Figure 4a depicts the monthly mean distribution of O_3_-8h-90per concentrations in NEC from 2013 to 2020. November, December, and January exhibited low O_3_-8h-90per concentrations with a monthly mean of approximately 70 μg/m^3^. 

The monthly mean began to increase in February, and the highest means were observed in central Liaoning, central Jilin, and eastern Heilongjiang, at approximately 90 μg/m^3^. Beginning in March, the monthly mean O_3_-8h-90per concentration increased considerably. It increased to 90 μg/m^3^ in NEC and to 100–110 μg/m^3^ in central Liaoning and Jilin. In April, the monthly mean increased further, and the highest concentration (approximately 130–140 μg/m^3^) was observed in central Liaoning. In May and June, the spatial distributions of the monthly mean O_3_-8h-90per concentrations were similar. The monthly mean O_3_-8h-90per concentrations in Liaoning increased considerably, and the highest value (170–180 μg/m^3^) was observed in central and western Liaoning, followed by central and western Jilin (140–150 μg/m^3^) and other regions (100–110 μg/m^3^). Beginning in July, the monthly mean O_3_-8h-90per concentrations and their distribution range gradually decreased in NEC. High monthly mean O_3_-8h-90per concentrations (approximately 160 μg/m^3^) were still observed in central and western Liaoning as well as central and western Jilin (130–140 μg/m^3^). In August, the highest monthly mean O_3_-8h-90per concentration in NEC was approximately 130–140 μg/m^3^ in central and western Liaoning and approximately 90–100 μg/m^3^ in Jilin and Heilongjiang. After September, the monthly mean O_3_-8h-90per concentrations in NEC began to decrease, and the monthly mean in northern Heilongjiang decreased more rapidly than in other regions. From September to October, the O_3_-8h-90per concentration in NEC continued to decrease, and the spatial distribution of low monthly concentrations gradually increased. The highest monthly mean O_3_-8h-90per concentrations were observed in central and western Liaoning, approximately 130 μg/m^3^. The O_3_-8h-90per concentrations in most parts of Jilin and Heilongjiang were approximately 90 μg/m^3^, and the lowest O_3_-8h-90per concentration (approximately 70 μg/m^3^) was observed in northern Heilongjiang. 

Figure 4b depicts the monthly mean distribution of PM_2.5_ concentrations in NEC. The PM_2.5_ concentrations in NEC were the highest in January, February, and December, and the highest monthly mean O_3_-8h-90per concentration was approximately 100 μg/m^3^. 

The highest PM_2.5_ concentration (approximately 100 μg/m^3^) in NEC was observed in January in central Liaoning and the center of the Harbin–Changchun urban agglomeration. In February, the PM_2.5_ concentration was lower than that in January but remained high at approximately 80–90 μg/m^3^. Beginning in March, the mean PM_2.5_ concentration in NEC exhibited a declining trend, with a mean PM_2.5_ concentration of approximately 60–70 μg/m^3^ and a higher mean PM_2.5_ concentration of approximately 80 μg/m^3^ in cities. In April, the monthly mean PM_2.5_ concentrations in NEC began to decline gradually, with approximately 50–60 μg/m^3^ observed in Liaoning and 40 μg/m^3^ observed in other regions. From May to September, the monthly mean PM_2.5_ concentration in NEC decreased to approximately 20 μg/m^3^. In October, the monthly mean PM_2.5_ concentrations in central Liaoning and central Jilin began to increase to approximately 60 μg/m^3^. The monthly mean PM_2.5_ concentration in NEC increased considerably in November, with the highest PM_2.5_ concentration (approximately 90 μg/m^3^) observed in central and western Liaoning, Jilin, and Heilongjiang. In December, the monthly mean PM_2.5_ concentration and its distribution range continued to increase in NEC. The highest PM_2.5_ concentrations were observed in central Liaoning and the Harbin–Changchun urban agglomeration, with the monthly mean PM_2.5_ concentration reaching 100 μg/m^3^.

The spatial distribution of these monthly variations in O_3_-8h-90per and PM_2.5_ concentrations revealed that O_3_-8h-90per and PM_2.5_ pollution in NEC exhibit unique and time-dependent distribution characteristics. December–February is the main period of PM_2.5_ pollution in NEC, whereas May–July is the main period of O_3_ pollution. Ma et al. (2021a) [50] investigated that the temperature could alter PM_2.5_ and O_3_ through both physical and chemical processes over the North China Plain.

### 3.4. Interannual Distribution and Trends of O_3_-8h-90per and PM_2.5_ in NEC

**Figure 5:**
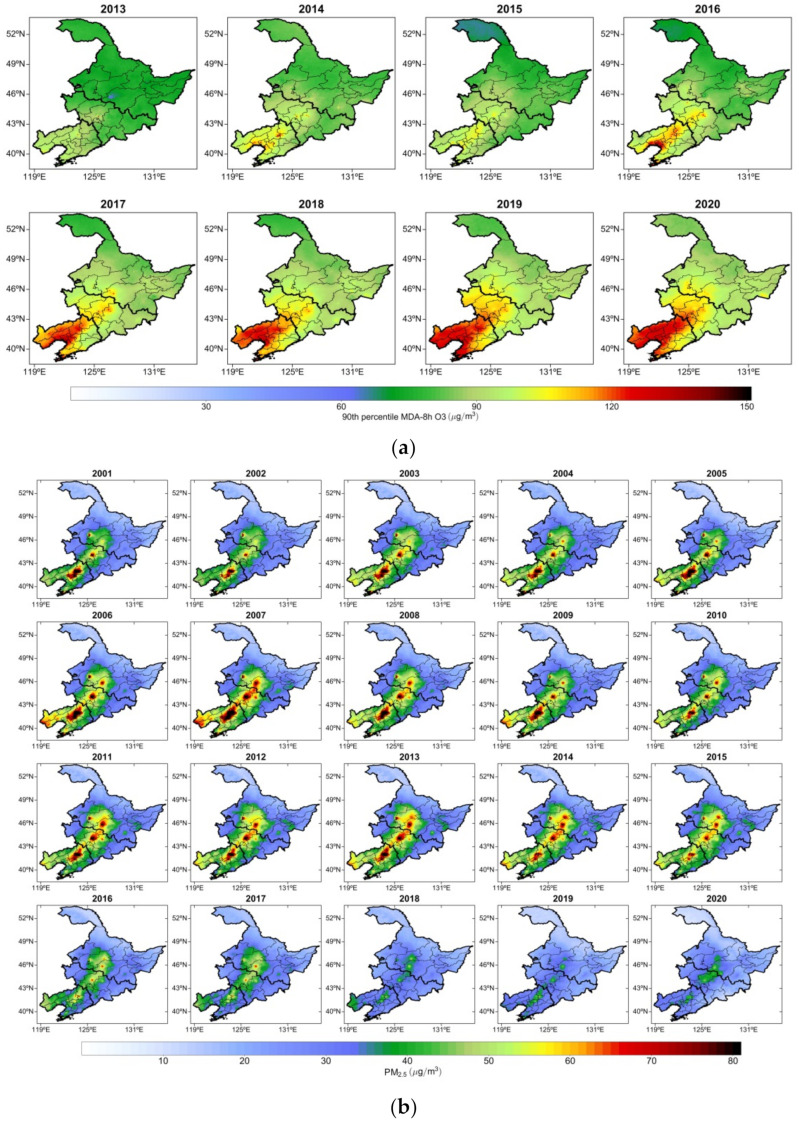
Spatial distribution of mean annual concentrations of (**a**) O_3_-8h-90per from 2013–2020 and (**b**) PM_2.5_ from 2001–2020 in NEC.

We analyzed the spatial distribution of the interannual means of O_3_-8h-90per concentration in NEC from 2013 to 2020 (Figure 5a). From 2013 to 2015, the annual mean O_3_-8h-90per concentrations in NEC were low (approximately 80–90 μg/m^3^), and the annual mean O_3_-8h-90per concentrations in typical areas in central Liaoning were relatively high (approximately 110–120 μg/m^3^). Beginning in 2016, the annual mean distribution of O_3_-8h-90per in Liaoning exhibited a considerable increasing trend, in terms of both concentration and regional distribution. In 2016, high O_3_-8h-90per concentrations in central Liaoning and the Bohai Rim area were observed mainly in the central regions, and the annual maximal O_3_-8h-90per concentration reached approximately 130 μg/m^3^. In the same period, the annual mean O_3_-8h-90per concentrations in central Jilin were also high. From 2017 to 2020, the spatial distribution of the high annual O_3_-8h-90per concentrations in Liaoning exhibited an expanding trend, and the annual mean O_3_-8h-90per concentration increased to 130–140 μg/m^3^ in western and northern Liaoning. At the same time, the annual mean O_3_-8h-90per concentrations in central and western Jilin increased to 110–120 μg/m^3^. Compared with Liaoning and Jilin, the spatial distribution of the annual mean O_3_-8h-90per concentrations in Heilongjiang exhibited only little change. The annual mean spatial distribution of O_3_-8h-90per concentration in Heilongjiang increased from 70–80 μg/m^3^ in 2013 to approximately 90–100 μg/m^3^ in 2020.

We analyzed the interannual variation in PM_2.5_ concentrations in NEC from 2001 to 2020 (Figure 5b). 

From 2001 to 2014, the spatial distribution and regional variation of PM_2.5_ concentrations in NEC were consistent. The high PM_2.5_ concentrations were distributed in a zonal pattern, with the highest value reaching 80 μg/m^3^, observed in the central Liaoning city cluster, central Jilin, and areas adjacent to southern Heilongjiang. In 2015, the areas in NEC with high PM_2.5_ exhibited a decreasing trend. These areas were concentrated in Liaoning, Jilin, Heilongjiang, and several typical cities, and the higher PM_2.5_ concentration exhibited a point-like distribution with values reaching 80 μg/m^3^. In 2016, the annual mean PM_2.5_ concentration in NEC decreased considerably, with the highest PM_2.5_ concentration, 50–60 μg/m^3^, distributed among Liaoning, Jilin, and central Heilongjiang. In 2017, the area exhibiting high PM_2.5_ concentrations decreased, and the highest PM_2.5_ concentrations (approximately 50 μg/m^3^) were observed in northern Liaoning as well as in central Jilin and Heilongjiang. In particular, from 2018 to 2020, the PM_2.5_ concentration in NEC decreased considerably and reached approximately 40 μg/m^3^ in typical cities such as Shenyang, Changchun, and Harbin. According to the analyzed data, the interannual characteristics of the spatial variation of O_3_-8h-90per and PM_2.5_ reveal that the O_3_-8h-90per concentration in NEC changed mainly regionally, exhibiting an increasing annual trend. By contrast, the PM_2.5_ concentration exhibited a zonal distribution characteristic from the southwest to the northeast, and the variation of PM_2.5_ concentrations decreased annually.

To determine the interannual variations of O_3_-8h-90per and PM_2.5_ concentrations in NEC, we investigated the monthly and annual variations of O_3_-8h-90per and PM_2.5_ concentrations in the three studied provinces. 

**Figure 6:**
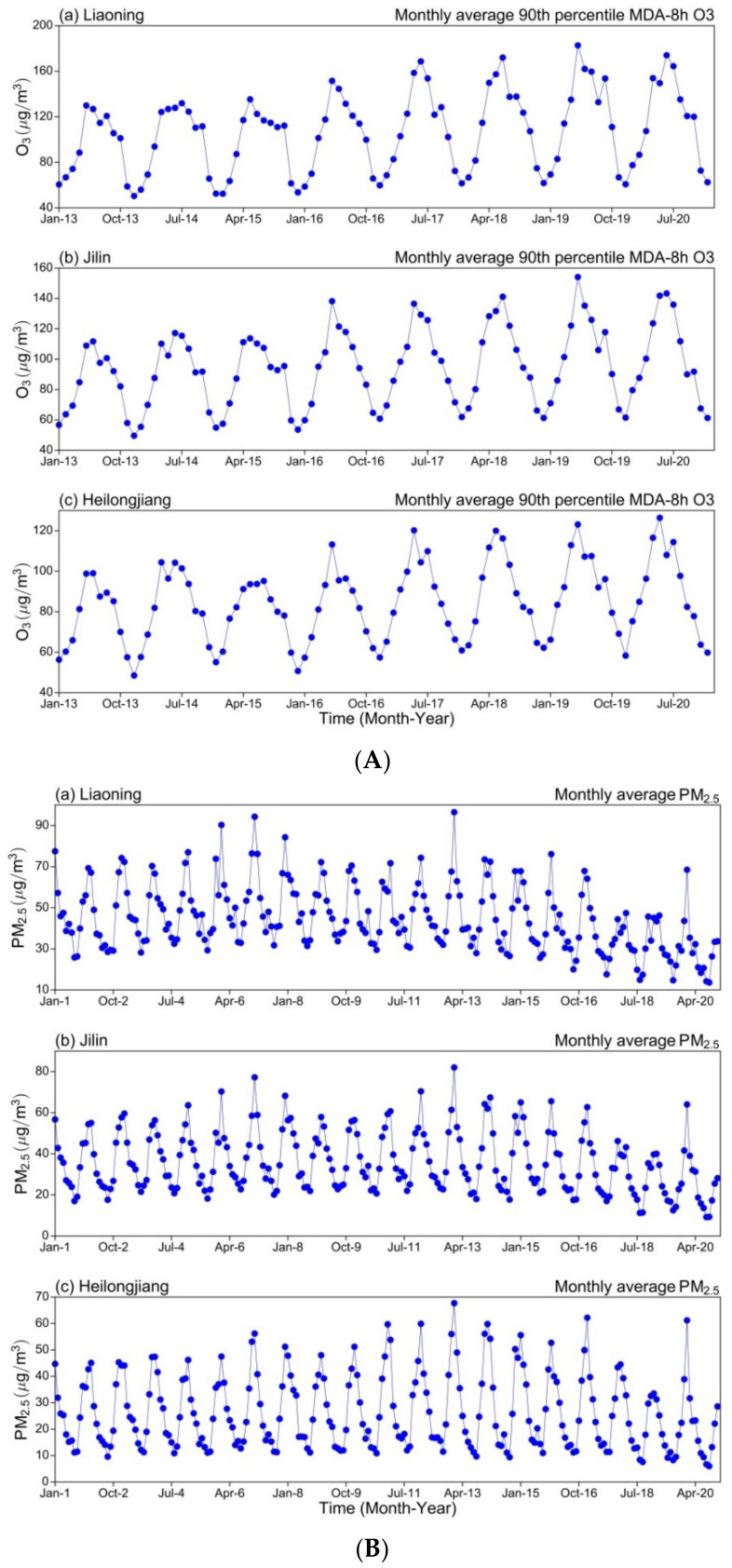
Interannual trend of mean monthly concentrations of (**A**) O_3_-8h-90per from 2013–2020 and (**B**) PM_2.5_ from 2001–2020 in (**a**) Liaoning, (**b**) Jilin and (**c**) Heilongjiang province over NEC.

As depicted in Figure 6A, the interannual variation of the monthly mean O_3_-8h-90per concentration in NEC exhibited obvious periodic variation during 2013–2020. The highest and lowest O_3_-8h-90per concentrations were observed from May to July and December to January, respectively. The monthly mean O_3_-8h-90per concentrations exhibited an increasing trend in Liaoning, Jilin, and Heilongjiang, respectively. By contrast, the interannual variation of the monthly mean PM_2.5_ concentration during 2001–2020 was not as substantial as the periodic variation of O_3_-8h-90per (Figure 6B). The highest PM_2.5_ concentration was observed in January and February, and the lowest was observed in August and September. The PM_2.5_ concentrations in Liaoning, Jilin, and Heilongjiang exhibited a small monthly decreasing trend, respectively. 

**Figure 7:**
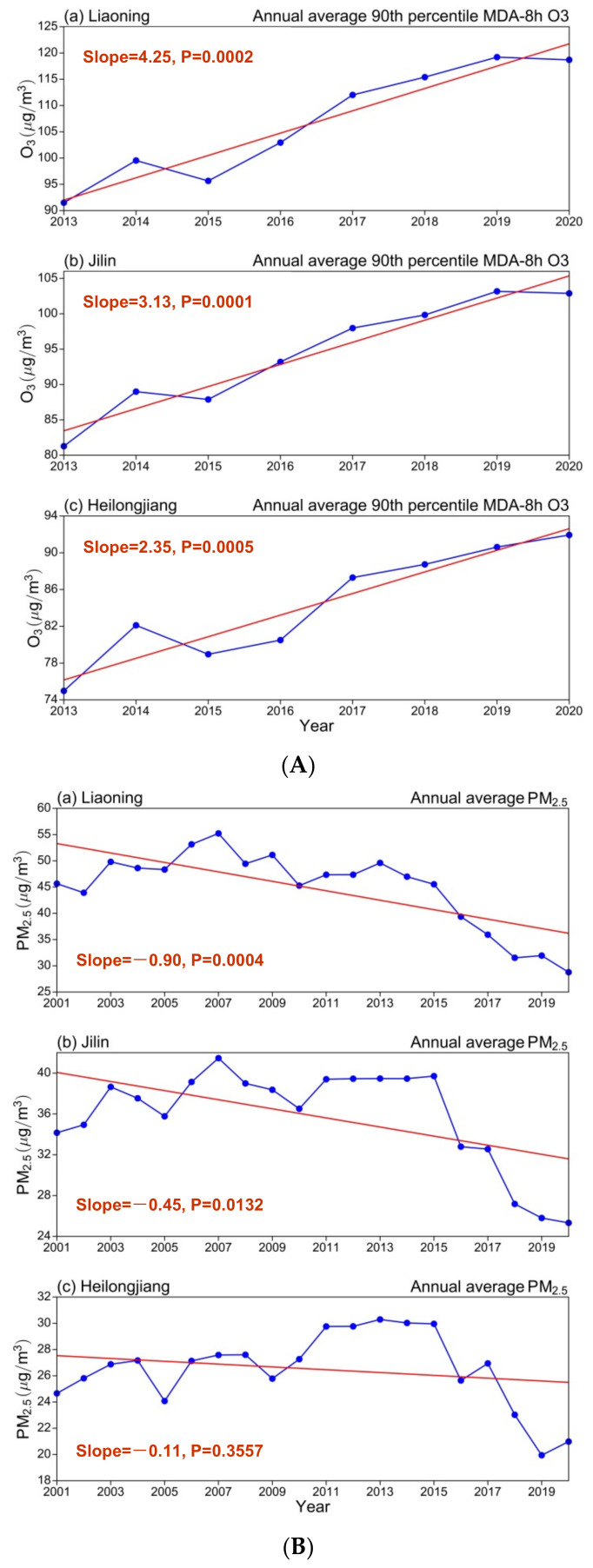
Interannual trend of mean annual concentrations of (**A**) O_3_-8h-90per from 2013–2020 and (**B**) PM_2.5_ from 2001–2020 in (**a**) Liaoning, (**b**) Jilin and (**c**) Heilongjiang province over NEC. Red solid lines indicate slopes with a linear trend and statistical significance at the 95% confidence level (*p* < 0.05).

We also investigated the trend in the annual mean variation of O_3_-8h-90per from 2013 to 2020 and PM_2.5_ concentrations from 2001 to 2020 in NEC. As depicted in Figure 7A, the annual mean increase of O_3_-8h-90per concentration exhibited slopes of 4.25, 3.13, and 2.35 (μg/m^3^)/yr in Liaoning, Jilin, and Heilongjiang, respectively. In 2013, the annual mean O_3_-8h-90per concentrations in Liaoning, Jilin, and Heilongjiang were low, approximately 91, 81, and 75 μg/m^3^, respectively; in 2014, they increased to approximately 100, 90, and 82 μg/m^3^; in 2015, they decreased to approximately 95, 87, and 78 μg/m^3^. In 2016, the annual mean O_3_-8h-90per concentrations in Liaoning, Jilin, and Heilongjiang increased rapidly to 120, 104, and 90 μg/m^3^, respectively, and continued increasing until 2019. In 2020, the annual mean O_3_-8h-90per concentrations decreased to approximately 117 μg/m^3^ in Liaoning and 102 μg/m^3^ in Jilin and increased to 92 μg/m^3^ in Heilongjiang.

We also investigated the interannual trend of PM_2.5_ concentrations in NEC from 2001 to 2020. As depicted in Figure 7B, the PM_2.5_ concentrations in Liaoning, Jilin, and Heilongjiang exhibited an annual declining trend with slopes of −0.90, −0.45, and −0.11 (μg/m^3^)/yr, respectively. 

From 2001 to 2005, the annual mean PM_2.5_ concentrations in Liaoning, Jilin, and Heilongjiang were relatively low, namely, 65, 50, and 38 μg/m^3^, respectively; the highest PM_2.5_ concentrations were approximately 75, 57, and 46 μg/m^3^, respectively, and were observed in 2003. In 2007, the PM_2.5_ concentrations increased to approximately 85, 70, and 50 μg/m^3^ in Liaoning, Jilin, and Heilongjiang, respectively. Subsequently, the PM_2.5_ concentrations in NEC exhibited a substantial increasing trend, with the annual mean concentrations increasing to 75, 65, and 58 μg/m^3^ in Liaoning, Jilin, and Heilongjiang, respectively, until 2013. After 2013, the PM_2.5_ concentration decreased considerably in NEC, and the annual minimal PM_2.5_ concentration was approximately 35 μg/m^3^ in Liaoning, Jilin, and Heilongjiang from 2018 to 2019. After 2020, the annual mean PM_2.5_ concentrations in the three provinces increased, with an annual mean of approximately 40–45 μg/m^3^. The time series of O_3_-8h-90per and PM_2.5_ in NEC indicates that the O_3_-8h-90per concentrations in the three provinces increased annually, whereas the PM_2.5_ concentration fluctuated and decreased. The inflection point of PM_2.5_ concentration change was observed in 2013. This might be related to air control policies that were implemented in NEC that year. As PM_2.5_ emissions decreased annually, the O_3_-8h-90per concentrations exhibited an increasing trend. This highlights the relevance of studying the mechanisms and interaction of PM_2.5_ that affect the formation of surface O_3_. Shao et al. (2021) [51] found that the significance of ozone enhancement due to PM_2.5_ dropping depends on both the PM_2.5_ levels and optical properties of particles in many mega-cities in China.

### 3.5. Interannual Variation and Trends of O_3_-8h-90per at the City Level in NEC

In this section, we present a comparison of city-level interannual variation in O_3_-8h-90per and PM_2.5_ concentrations in 35 cities. In Liaoning, the following 14 cities were included: Dandong, Fushun, and Benxi in the east, Jinzhou, Huludao, Fuxin, and Chaoyang in the west, Yingkou, Dalian, and Panjin in the south, Tieling in the north, and Shenyang, Liaoyang, and Anshan in central Liaoning. In Jilin, the following nine cities were included: Yanbian and Baishan in the east, Baicheng, Songyuan, and Siping in the west, Tonghua in the south, and Changchun, Jilin, and Liaoyuan in central Jilin. In Heilongjiang, the following 12 cities were included: Yichun, Qitaihe, Mudanjiang, Jiamusi, Shuangyashan, Hegang, Jixi, Qiqihar, and Daqing in the west, Harbin in the south, Heihe in the north, and Suihua in central Heilongjiang. 

**Figure 8:**
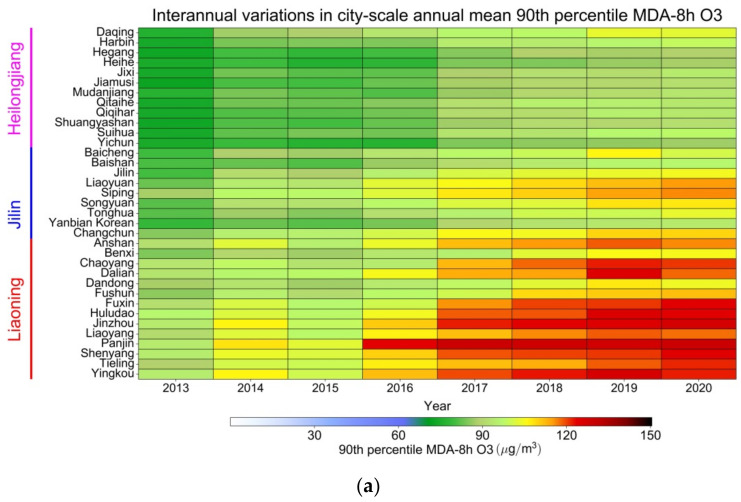

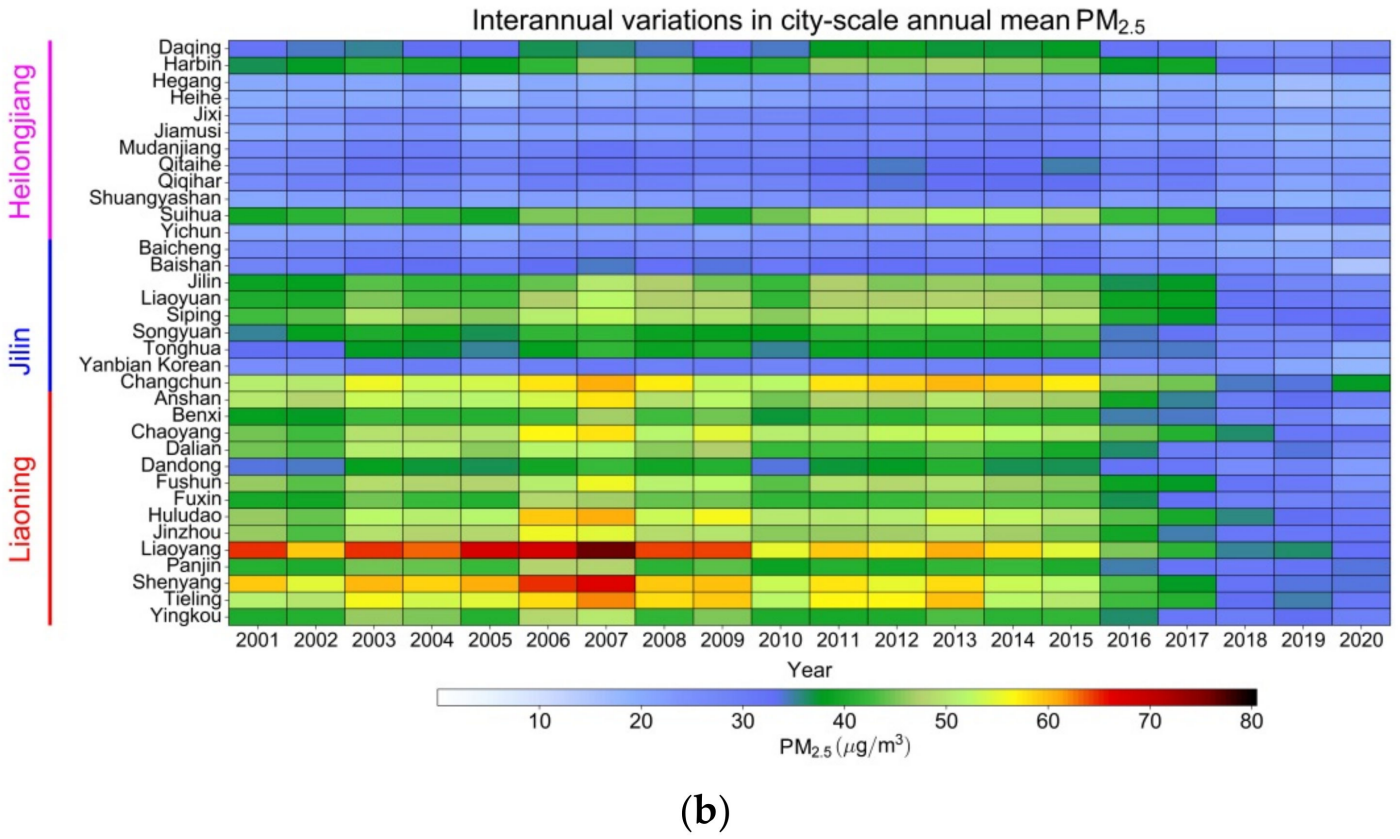
Interannual trends of (**a**) O_3_-8h-90per from 2013–2020 and (**b**) PM_2.5_ from 2001–2020 in 35 cities of NEC.

As depicted in Figure 8a, the interannual variations in O_3_-8h-90per concentrations in different cities exhibited distinct trends in increases and increase ranges. 

From 2013 to 2015, the annual fluctuation variation in O_3_-8h-90per concentrations in different cities in Liaoning was small, and the concentration was approximately 90–110 μg/m^3^. In 2016, the annual mean O_3_-8h-90per concentration in Panjin, Yingkou, and Dalian (southern Liaoning) began to increase considerably, whereas the O_3_-8h-90per concentration remained at 120–130 μg/m^3^. Jinzhou, Huludao, Fuxin, and Chaoyang (western Liaoning) and Tieling (northern Liaoning) exhibited the next-highest increase and an O_3_-8h-90per concentration of approximately 120 μg/m^3^. The O_3_-8h-90per concentration in Shenyang, Liaoyang, and Anshan (central Liaoning) was approximately 100 μg/m^3^. The lowest O_3_-8h-90per concentration was observed in Dandong, Fushun, and Benxi (eastern Liaoning) with a concentration of approximately 90–100 μg/m^3^ in 2019–2020. The O_3_-8h-90per concentrations in cities in Jilin were lower than those in Liaoning. From 2013 to 2015, the annual flat variation in O_3_-8h-90per concentrations in Jilin was small, and the concentration was approximately 80 μg/m^3^. In 2016, the O_3_-8h-90per concentration in Changchun, Jilin, and Liaoyuan (central Jilin) began to increase to approximately 110 μg/m^3^. Similarly, higher O_3_-8h-90per concentrations (100 μg/m^3^) were recorded in Baicheng, Songyuan, and Siping (western Jilin). The O_3_-8h-90per concentration in Tonghua (southern Jilin) was approximately 90 μg/m^3^ from 2019 to 2020. The O_3_-8h-90per concentration (approximately 80 μg/m^3^) was rather low in Yanbian and Baishan (eastern Jilin). The city-level O_3_-8h-90per concentration in Heilongjiang was lower than that in Liaoning and Jilin. From 2013 to 2015, the annual flat variation in O_3_-8h-90per concentrations in Heilongjiang was approximately 70–80 μg/m^3^. In 2016, the O_3_-8h-90per concentration exhibited an overall small increase. The highest O_3_-8h-90per concentration was approximately 100 μg/m^3^ in Daqing (western Heilongjiang). The O_3_-8h-90per concentration was approximately 90 μg/m^3^ in Harbin and Suihua (western and central Heilongjiang, respectively). The annual mean variation in O_3_-8h-90per concentrations in other areas of Heilongjiang from 2019 to 2020 was approximately 80 μg/m^3^.

These results reveal that the distribution of high city-level O_3_-8h-90per concentrations in NEC was consistent with the spatial distribution of ground temperature. Under the combined influence of high temperatures and solar radiation, the O_3_ formation is enhanced. In NEC, the mean temperature in various cities was closely related to distinct meteorological factors, such as the location of land and sea, latitude, topography, and altitude. Studies have reported that rising temperatures could be related to high O_3_ pollution in NEC [52,53]. The temperature in Liaoning increased from east to west and from north to south. Therefore, the O_3_-8h-90per concentrations in Huludao, Panjin, and Dalian (southwestern Liaoning) were high, whereas those in Fushun and Benxi (eastern Liaoning) were low. A similar temperature difference between east and west was observed in Jilin. The temperature was relatively high in western Jilin, especially in Baicheng and Siping (southwest Jilin); this high temperature was conducive to the formation of O_3_-8h-90per. The temperature in Baishan and Yanbian (eastern Jilin) was relatively low; therefore, the O_3_-8h-90per concentration was low. The temperature was relatively high in southern Heilongjiang; therefore, the cities with high O_3_-8h-90per concentration were distributed mostly in southern Heilongjiang. The northern cities exhibited low O_3_-8h-90per concentrations.

The city-level trends of PM_2.5_ in NEC were opposite to those of O_3_-8h-90per, and several cities exhibited a considerable decrease in PM_2.5_ (Figure 8b).

From 2001 to 2015, the annual PM_2.5_ concentrations in different regions of Liaoning exhibited high variation, and the highest PM_2.5_ concentration was approximately 70 μg/m^3^ in Shenyang, Liaoyang, and Anshan (central Liaoning). This was followed by that in Tieling (northern Liaoning), with a maximum PM_2.5_ concentration of approximately 60–70 μg/m^3^. In Jinzhou, Huludao, Fuxin, and Chaoyang (western Liaoning), the highest PM_2.5_ concentration decreased to 60 μg/m^3^. In Dandong, Fushun, and Benxi (eastern Liaoning) as well as Yingkou, Dalian, and Panjin (southern Liaoning), the PM_2.5_ concentration was approximately 35–55 μg/m^3^. In 2016, the city-level PM_2.5_ concentration in Liaoning began to decrease to approximately 25–35 μg/m^3^ until 2020. From 2001 to 2015, the city-level PM_2.5_ concentrations in some areas of Jilin were relatively high. The highest PM_2.5_ concentration in Jilin was observed in Changchun (central Jilin) at approximately 60 μg/m^3^. Siping and Liaoyuan (western and central Jilin, respectively) exhibited a PM_2.5_ concentration of approximately 55 μg/m^3^. Low PM_2.5_ concentrations (approximately 20 μg/m^3^) were observed in Yanbian and Baishan (eastern Jilin) and Tonghua (southern Jilin). The overall city-level PM_2.5_ concentration in Heilongjiang was lower than that in Liaoning and Jilin. From 2013 to 2015, the annual mean PM_2.5_ concentrations (approximately 50 μg/m^3^) were relatively large in Suihua and Harbin (central Heilongjiang). Daqing (western Heilongjiang) exhibited the next-highest value of 40 μg/m^3^. Qitaihe (eastern Heilongjiang) and Qiqihar (western Heilongjiang) exhibited a PM_2.5_ concentration of approximately 35 μg/m^3^. In 2016, the PM_2.5_ concentration decreased overall, and in 2019–2020, it was approximately 20–30 μg/m^3^.

Compared with the changes in O_3_-8h-90per concentration, the interannual variation of PM_2.5_ in NEC revealed PM_2.5_ pollution at the city level. The cities with high PM_2.5_ concentrations were characterized by industrial activity and large populations. Therefore, the reduction of environmental pollution caused a reduction in the contribution of PM_2.5_ to air pollution at the city level in NEC.

**Figure 9:**
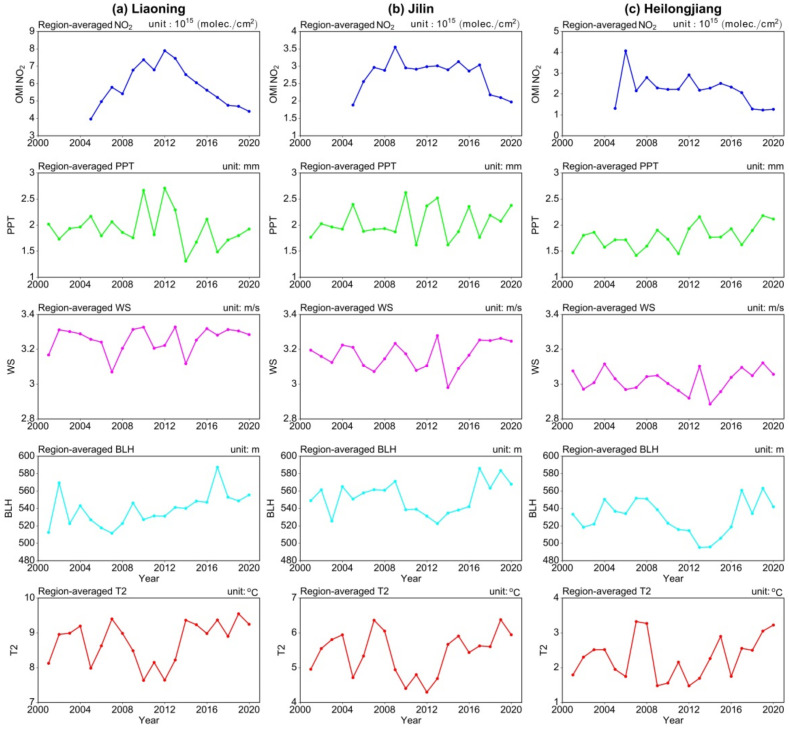
Interannual variation of region-averaged tropospheric NO_2_ columns, precipitation (PPT), wind speed (WS), boundary layer height (BLH), and temperature at 2 m (T2) in (**a**) Liaoning, (**b**) Jilin, and (**c**) Heilongjiang in NEC. Note that tropospheric NO_2_ columns data are available for the years 2005–2020.

In order to discuss the potential impact of important meteorological factors and emission sources on O_3_-8h-90per and PM_2.5_, we further analyzed the annual variation of tropospheric NO_2_ column from 2005 to 2020, precipitation (PPT), wind speed (WS), boundary layer height (BLH), and temperature at 2 m (T2) from 2001 to 2020 in NEC. Figure 9 shows that the tropospheric NO_2_ column in NEC has been decreasing since 2012, which is consistent with the decreasing trend of the interannual variation of PM_2.5_, indicating the impact of the decreasing intensity of anthropogenic emission sources on PM_2.5_ concentration. From the interannual variation of meteorological elements in NEC, the higher wind speed and the increase of BLH are conducive to the diffusion of PM_2.5_ to a certain extent. It is worth noting that temperature at 2 m has shown a significant increasing trend since 2012. In addition to the increase of precursors, the rise of surface temperature may be the main meteorological driving factor affecting the O_3_-8h-90per concentration. In addition to emission and meteorological factors, the chemical mechanism affecting O_3_ and PM_2.5_ is also important and needs further study.

## 4. Discussion

With increasing urbanization, environmental air quality has become an increasingly critical public concern. The pollution caused by PM_2.5_ and O_3_-8h-90per has considerably affected people and residential environments. In almost ten years, surface O_3_-8h-90per and PM_2.5_ pollution has become an environmental concern. Therefore, studying the distribution and variation of surface O_3_ and PM_2.5_ concentrations has high theoretical and practical value and aids in further investigating the meteorological and chemical factors that determine regional pollution. From the perspective of formation mechanism and meteorology, many previous studies have conducted important research on the variation characteristics of O_3_ and PM_2.5_ concentration in different regions of China. Zhao et al. (2018) [54] pointed out that the PM_2.5_ could directly transport from one city to another city, while Tibetan Plateau may be an important source region of high ozone in Sichuan Basin of southwest China. Ma et al. (2021b) [55] illustrated that the increase in volatile organic compounds (VOCs) along with depletions in NO_2_ and CO significantly boosted the ozone photochemical production in North China plain. Wang et al. (2021) [56] showed that the annual summertime ozone over Central China was significantly correlated with the springtime thermal forcing, indicated by total atmospheric energy over Tibetan Plateau in an interdecadal timescale. During the COVID-19 lockdown period, the results of Yin et al. (2021) [57] suggest that conventional emission reduction of NO_x_ could not be sufficient to reduce surface O_3_ concentration, and ozone pollution needs to be controlled by a variety of pollutants in central China. The significance of this study is to improve the scientific understanding of multi-year changes of O_3_-8h-90per and PM_2.5_ concentrations due to climatological characteristics in NEC. This paper focuses on the O_3_-8h-90per and PM_2.5_ pollution at different temporal and spatial scales, especially at city level in NEC. These results could provide reference on the formation mechanism of ozone and PM_2.5_ in NEC for further study. Moreover, the correlation between O_3_ and PM_2.5_ is related to the reactions with VOCs. Ozone could oxidize VOCs to less volatile products that likely partition to the particle phase. Therefore, collaborative control of O_3_ and PM_2.5_ must be accelerated to reduce regional haze pollution and photochemical smog events.

## 5. Conclusions

In this study, TAP O_3_-8h-90per and PM_2.5_ concentration data were used to analyze the multi-year variation of O_3_-8h-90per and PM_2.5_ concentrations at distinct temporal and spatial scales in NEC.

The concentrations of O_3_-8h-90per were highest in northern, western, and southern Liaoning and the Bohai Rim (up to 120 μg/m^3^). The highest O_3_-8h-90per concentrations (approximately 90–100 μg/m^3^) in Jilin were distributed in the central and western regions. The O_3_-8h-90per concentration in Heilongjiang was relatively small (approximately 80 μg/m^3^). The highest concentrations of PM_2.5_ were observed in Shenyang, Changchun, Harbin, and other major provincial cities in NEC (approximately 60 μg/m^3^). The spatial distribution of high PM_2.5_ concentrations was inconsistent with that of high O_3_-8h-90per concentrations. The spatial distribution of O_3_-8h-90per and PM_2.5_ was closely related to the meteorological factors, population density, and environmental impact. In addition, O_3_ could also oxidize pollutants in the air and increase PM_2.5_, while PM_2.5_ can reduce the solar radiation reaching the ground and decrease the rate of O_3_ photochemical formation.

The O_3_-8h-90per concentrations were highest in spring and summer, followed by autumn and winter. In spring, the highest O_3_-8h-90per concentrations (approximately 120–130 μg/m^3^) were observed in central and western Liaoning. Then, in summer, the O_3_-8h-90per concentration increased considerably in the whole province (to approximately 150 μg/m^3^). In autumn, the O_3_-8h-90per concentration in NEC decreased markedly until, in winter, the O_3_-8h-90per concentration reached one of its lowest values (approximately 60 μg/m^3^). By contrast, the distribution of PM_2.5_ concentration increased considerably in winter, followed by that in spring and autumn; the lowest PM_2.5_ concentration was observed in summer. In spring, the highest PM_2.5_ concentrations (approximately 60 μg/m^3^) were observed in central Liaoning and central Jilin. In summer, the PM_2.5_ concentration in NEC decreased. Then, in autumn and winter, the PM_2.5_ concentration in central Liaoning increased considerably, reaching approximately 80–90 μg/m^3^. The highest PM_2.5_ concentrations in winter were affected mainly by emissions, whereas meteorological conditions such as high temperatures and strong solar radiation were conducive to the formation of O_3_.

The monthly mean O_3_-8h-90per concentration was approximately 70 μg/m^3^, with low concentrations observed in November, December, and January. In May and June, the spatial distribution of the monthly mean O_3_-8h-90per concentration increased substantially, and the highest O_3_-8h-90per concentrations were observed in central and western Liaoning (170–180 μg/m^3^), followed by central and western Jilin (140–150 μg/m^3^) and other regions (100–110 μg/m^3^). By contrast, the PM_2.5_ concentrations (approximately 100 μg/m^3^) were higher in December, January, and February. The highest PM_2.5_ concentration was observed in January in central Liaoning and the central part of the Harbin–Changchun urban agglomeration; the highest PM_2.5_ concentration was approximately 100 μg/m^3^. In February, the PM_2.5_ concentration was lower than that of January, but remained high (approximately 80–90 μg/m^3^).

From 2013 to 2015, the annual mean O_3_-8h-90per concentration was low (approximately 80–90 μg/m^3^). In 2016, the annual mean distribution of O_3_-8h-90per in Liaoning began to considerably increase, both in concentration level and in regional distribution. From 2001 to 2014, the highest PM_2.5_ concentrations were distributed in a zonal pattern, with the highest concentrations (80 μg/m^3^) observed in central Liaoning, central Jilin, and the areas adjacent to southern Heilongjiang. In 2015, the PM_2.5_ concentrations in Liaoning, Jilin, Heilongjiang, and other typical cities exhibited a decreasing trend, reaching approximately 80 μg/m^3^. The interannual variation of the monthly mean O_3_-8h-90per concentrations exhibited a marked periodic variation during 2013–2020. The monthly mean O_3_-8h-90per concentrations in Liaoning, Jilin, and Heilongjiang exhibited an increasing trend, while the PM_2.5_ concentrations showed a small monthly decreasing trend, respectively. The annual mean increases of O_3_-8h-90per concentration in the three provinces exhibited slopes of 4.25, 3.13, and 2.35 (μg/m^3^)/yr in Liaoning, Jilin, and Heilongjiang, respectively, and the PM_2.5_ concentrations in Liaoning, Jilin, and Heilongjiang exhibited annual decreasing trends with slopes of −0.90, −0.45 and −0.11 (μg/m^3^)/yr, respectively.

In 2016, the annual mean O_3_-8h-90per concentrations in Panjin, Yingkou, and Dalian (southern Liaoning) began to increase markedly (120–130 μg/m^3^). In 2016, the O_3_-8h-90per concentrations in Changchun, Jilin, and Liaoyuan (central Jilin) were relatively high (approximately 110 μg/m^3^). From 2013 to 2015, the annual flat variation of O_3_-8h-90per concentration in Heilongjiang was approximately 70–80 μg/m^3^. The annual mean variation in O_3_-8h-90per concentrations in other areas of Heilongjiang from 2019 to 2020 was approximately 80 μg/m^3^. The temperature distribution in Liaoning increased from east to west and from north to south. The city-level distribution of high O_3_-8h-90per concentrations was consistent with the spatial distribution of ground temperatures.

From 2001 to 2015, the annual PM_2.5_ concentration in various regions of Liaoning exhibited high variation. In 2016, the city-level PM_2.5_ concentration in Liaoning began to decrease, reaching approximately 25–35 μg/m^3^ in 2020. From 2001 to 2015, the city-level PM_2.5_ concentrations in some areas of Jilin were high. From 2013 to 2015, the annual mean PM_2.5_ concentrations were relatively high in Suihua and Harbin (central Heilongjiang), approximately 50 μg/m^3^; Subsequently, the PM_2.5_ concentration decreased overall, reaching approximately 20–30 μg/m^3^ in 2019–2020. The cities with high PM_2.5_ concentration were characterized by industrial activity and high population densities. Since 2012, the interannual variation of tropospheric NO_2_ has been decreasing, which is consistent with PM_2.5_, indicating the influence of anthropogenic emission sources on the level of PM_2.5_; on the contrary, the temperature at 2 m showed a significant increasing trend, which had an important impact on the increase of O_3_-8h-90per concentration The objective of this study was to elucidate the multi-year variation and current levels of O_3_ and PM_2.5_ pollution in NEC and provide crucial scientific support for regional air pollution prevention and control measures in China.

## Data Availability

The datasets generated during and/or analyzed during the current study are available from the corresponding author on reasonable request.
